# Increased Drp1-Mediated Mitochondrial Fission Promotes Proliferation and Collagen Production by Right Ventricular Fibroblasts in Experimental Pulmonary Arterial Hypertension

**DOI:** 10.3389/fphys.2018.00828

**Published:** 2018-07-10

**Authors:** Lian Tian, Francois Potus, Danchen Wu, Asish Dasgupta, Kuang-Hueih Chen, Jeffrey Mewburn, Patricia Lima, Stephen L. Archer

**Affiliations:** Department of Medicine, Queen’s University, Kingston, ON, Canada

**Keywords:** mitochondrial fission, mitochondrial dynamics, dynamin-related protein 1 (Drp1), fibrosis, mitochondrial division inhibitor 1 (Mdivi-1), P110

## Abstract

**Introduction:** Right ventricular (RV) fibrosis contributes to RV failure in pulmonary arterial hypertension (PAH). The mechanisms underlying RV fibrosis in PAH and the role of RV fibroblasts (RVfib) are unknown. Activation of the mitochondrial fission mediator dynamin-related protein 1 (Drp1) contributes to dysfunction of RV myocytes in PAH through interaction with its binding partner, fission protein 1 (Fis1). However, the role of mitochondrial fission in RVfib and RV fibrosis in PAH is unknown.

**Objective:** We hypothesize that mitochondrial fission is increased in RVfib of rats with monocrotaline (MCT)-induced PAH. We evaluated the contribution of Drp1 and Drp1–Fis1 interaction to RVfib proliferation and collagen production in culture and to RV fibrosis *in vivo*.

**Methods:** Vimentin (+) RVfib were enzymatically isolated and cultured from the RVs of male Sprague–Dawley rats that received MCT (60 mg/kg) or saline. Mitochondrial morphology, proliferation, collagen production, and expression of Drp1, Drp1 binding partners and mitochondrial fusion mediators were measured. The Drp1 inhibitor mitochondrial division inhibitor 1 (Mdivi-1), P110, a competitive peptide inhibitor of Drp1–Fis1 interaction, and siRNA targeting Drp1 were assessed. Subsequently, prevention and regression studies tested the antifibrotic effects of P110 (0.5 mg/kg) *in vivo*. At week 4 post MCT, echocardiography and right heart catheterization were performed. The RV was stained for collagen.

**Results:** Mitochondrial fragmentation, proliferation rates and collagen production were increased in MCT-RVfib versus control-RVfib. MCT-RVfib had increased expression of activated Drp1 protein and a trend to decreased mitofusin-2 expression. Mdivi-1 and P110 inhibited mitochondrial fission, proliferation and collagen III expression in MCT-RVfib. However, P110 was only effective at high doses (1 mM). siDrp1 also reduced fission in MCT-RVfib. Despite promising results in cell therapy, *in vivo* therapy with P110 failed to prevent or regress RV fibrosis in MCT rats, perhaps due to failure to achieve adequate P110 levels or to the greater importance of interaction of Drp1 with other binding partners.

**Conclusion:** PAH RVfib have increased Drp1-mediated mitochondrial fission. Inhibiting Drp1 prevents mitochondrial fission and reduces RVfib proliferation and collagen production. This is the first description of disordered mitochondrial dynamics in RVfib and suggests that Drp1 is a potential new antifibrotic target.

## Introduction

Pulmonary arterial hypertension (PAH) is characterized by pulmonary vascular obstruction, vascular stiffening and vasoconstriction, leading to increased right ventricular (RV) afterload and, consequently, right ventricular hypertrophy (RVH). Ultimately, pulmonary vascular disease leads to death from RV failure. Despite the importance of pulmonary vascular hemodynamics in PAH, RV function is the major determinant of the long-term prognosis (D’Alonzo et al., 1991; Sandoval et al., 1994; Campo et al., 2010; Ghio et al., 2010; Humbert et al., 2010; Sachdev et al., 2011; Voelkel et al., 2015). Some PAH patients, such as those with Eisenmenger’s syndrome, respond to increased afterload with an adaptive form of RVH, which is associated with a good prognosis; whereas, others, such as patients with scleroderma, have a maladaptive form of RVH. While the differences between adaptive and maladaptive RVH remain poorly defined, patients with maladaptive RVH have worse functional capacity. These patients have greater impairment of angiogenesis, adrenergic signaling and metabolism, and display impaired RV morphology characterized by RV dilatation and fibrosis (Archer et al., 2013; Ryan and Archer, 2014).

Disorders of mitochondrial metabolism, notably an increase in uncoupled glycolysis due to activation of pyruvate dehydrogenase kinase (the Warburg phenomenon), contribute to impaired RV myocyte function in RVH (Piao et al., 2010, 2013; Fang et al., 2012). The Warburg phenomenon also promotes an hyperproliferative, apoptosis-resistant phenotype in pulmonary artery smooth muscle cells (PASMC) in PAH (Michelakis et al., 2002; McMurtry et al., 2004; Bonnet et al., 2006). A similar Warburg shift in metabolism in pulmonary adventitial fibroblasts, mediated by epigenetic changes in pyruvate kinase muscle isoform 2/isoform 1 ratio, contributes to a hyperproliferative, profibrotic vascular fibroblast phenotype in PAH (Zhang et al., 2017).

In addition to metabolic changes (Sutendra and Michelakis, 2014), the mitochondria in PAH display structural changes due to disorders of mitochondrial dynamics that are linked to cell cycle regulation (Marsboom et al., 2012) and production of reactive oxygen species (Tian et al., 2017). Mitochondria undergo dynamic cycles of fission (division) and fusion (union) to form a highly plastic network. The mitochondrial network is regulated by various GTPase, including the fusion mediators mitofusin-1 (MFN1), mitofusin-2 (MFN2), and optic atrophy-1 (OPA1) and the fission mediator, dynamin-related protein 1 (Drp1) (Chen et al., 2003; Westermann, 2010). Inactivated Drp1 resides in the cytosol. Once activated by either dephosphorylation at Serine 637 (Cereghetti et al., 2008; Sharp et al., 2014, 2015), phosphorylation at Serine 616 (Taguchi et al., 2007; Marsboom et al., 2012), or both (Rehman et al., 2012), Drp1 translocates to the outer mitochondrial membrane where it associates with one or more of its binding partners, including fission protein 1 (Fis1), mitochondrial fission factor (MFF), and mitochondrial dynamics proteins of 49 and 51 kDa (MiD49 and MiD51) (Otera et al., 2010; Zhao et al., 2011; Loson et al., 2013; Chen et al., 2018). Activated Drp1 and its binding partners multimerize, forming a ring-like structure, which constricts and divides the mitochondrion, resulting in mitochondrial fission (Lee et al., 2004; Zhu et al., 2004; Chan, 2007; Youle and van der Bliek, 2012; Archer, 2013). Increased rates of mitochondrial fission are observed in PASMC in PAH, resulting in a fragmented mitochondrial network that promotes a hyperproliferative, apoptosis-resistant phenotype (Bonnet et al., 2007; Marsboom et al., 2012). This increase in mitotic fission is coordinated with cell cycle progression and reflects a shared reliance of fission and mitosis on certain kinases, including cyclin B1-CDK1 (Marsboom et al., 2012). However, the importance of increased fission is contextual and varies by cell type. For example, excessive mitochondrial fission in RV myocytes in PAH leads to increased production of mitochondrial-derived reactive oxygen species and impaired RV diastolic function (Tian et al., 2017), rather than changes in cell proliferation.

Inhibiting mitochondrial fission by targeting Drp1 has therapeutic potential in PAH. Mitochondrial division inhibitor 1 (Mdivi-1), a selective Drp1 GTPase activity inhibitor (Cassidy-Stone et al., 2008), inhibits mitochondrial fission and reduces proliferation in PASMC from PAH patients and improves hemodynamics *in vivo* in animal models of pulmonary hypertension (Marsboom et al., 2012). Mdivi-1 also inhibits mitochondrial fission in rat left ventricular (LV) myocytes and improves LV function both in *ex vivo* Langendorff ischemia-reperfusion injury model in rat and in *in vivo* cardiac arrest model in mouse (Sharp et al., 2014, 2015). P110, a relatively novel drug, is a 7-amino acid peptide representing a homology sequence between Drp1 and Fis1 (Guo et al., 2013; Qi et al., 2013). It is delivered across cell membranes and can cross the blood**–**brain barrier with the TAT_47_**_-_**_57_ carrier peptide (Guo et al., 2013). P110 selectively inhibits pathological, but not physiological mitochondrial fission (Guo et al., 2013; Qi et al., 2013). Blocking the interaction between Drp1 and Fis1 with P110 also preserves mitochondrial morphology and cellular function in rat cardiac myocytes under ischemia-reperfusion injury *in vitro* and *ex vivo* and improves LV function in an ischemia-reperfusion injury model *in vivo* (Disatnik et al., 2013). Our group has also demonstrated that P110 improves mitochondrial function and preserves RV diastolic function in both normal and monocrotaline (MCT)-induced PAH rat RVs in ischemia-reperfusion injury model using the Langendorff preparation (Tian et al., 2017). However, Fis1 is not important to the increased fission observed in PAH PASMC (Chen et al., 2018). The mitochondrial metabolic and mitochondrial dynamics profile of RV fibroblasts (RVfib) is unknown, as its potential relevance to RV fibrosis.

Here, we isolated RVfib from normal and pulmonary hypertensive rats and studied changes in mitochondrial dynamics. We focused on these cells as they are likely the major determinant of the RV fibrosis that occurs in maladaptive RVH. We compared RVfib from control versus monocrotaline (MCT)-induced PAH rats, using this well-established model because of the predisposition of the MCT RV to develop fibrosis and the MCT rat to die of RV failure. We characterized the mitochondrial dynamics in RVfib and examined the relationship between the observed increase in mitochondrial fission and increased rates of RVfib proliferation and collagen production. We then examined the effects of Mdivi-1 and P110 on mitochondrial morphology, cell proliferation and collagen production. We also evaluated the role of Drp1–Fis1 interaction in the regulation of mitochondrial fission in RVfib in PAH.

We demonstrate that MCT-RVfib have a fragmented mitochondrial phenotype due to excessive mitochondrial fission mediated by Drp1 activation. This phenotype persists in culture, suggesting it may be epigenetically mediated. This increase in fission promotes excess proliferation and collagen production. The Drp1 inhibitor Mdivi-1, small inhibitory RNA targeting Drp1 (siDrp1), and high doses of P110 each reverse mitochondrial fission. Both Mdivi-1 and high dose P110 reduce proliferation and collagen production in MCT-RVfib *in vitro*. Despite promising results in cell culture, P110 was not effective *in vivo*, at the administered dose. This is the first description of increased mitochondrial fission as a mediator of cardiac fibroblast proliferation and collagen production. Drp1 is a potential new antifibrotic target in PAH.

## Materials and Methods

Experiments were conducted in accordance with the published guidelines of the Canadian Council on Animal Care and approved by the Queen’s University Animal Care Committee.

### Reagents

Monocrotaline (MCT; C2401), Mdivi-1 (M0199), dimethyl sulfoxide (DMSO; D2650), collagenase (C0130), Dulbecco’s modified Eagle’s medium (DMEM; D5796), 10% neutral buffered formalin (HT501128), and bovine serum albumin (A7906) were purchased from Sigma (St. Louis, MO, United States). Both P110 and the peptide control sequence, TAT, were purchased from United Peptide (Herndon, VA, United States). L-glutamine (25030081), fetal bovine serum (SH3039603), penicillin-streptomycin (15140163), trypsin-EDTA (25200056), Hanks’ Balanced Salt Solution (SH3058802), phosphate-buffered saline (PBS; SH3025601), paraformaldehyde (AC416780250), Triton X-100 (BP151-100), and Tween-20 (BP337-100) were purchased from Thermo Fisher Scientific (Waltham, MA, United States). Fibroblast growth factor-basic was purchased from ProSpec (CYT-608; East Brunswick, NJ, United States).

### Monocrotaline-Induced PAH Animal Model

Male Sprague–Dawley rats (∼270 g) (Charles River, QC, Canada) received a single subcutaneous injection of monocrotaline (MCT; 60 mg/kg) (*n* = 45) or PBS (*n* = 5). We assessed the effects of P110 both as a prevention and as a regression intervention. In both protocols, P110 and TAT were administered at 0.5 mg/kg via intraperitoneal injection. In the prevention group (MCT-P3 group; *n* = 5), P110 was injected once at the time of injection of MCT.

There were two regression protocols. In one, P110 (MCT-P1 group; *n* = 14) or TAT (MCT-T1 group; *n* = 14) was injected on day 14 and 19 post MCT injection. In the other, P110 (MCT-P2 group; *n* = 7) or TAT (MCT-T2 group; *n* = 5) was injected on alternating days beginning day-10 post MCT injection.

### Echocardiography

At week 4 after MCT injection, Doppler, 2-dimensional, and M-mode echocardiography was performed using a high-frequency ultrasound system (Vevo 2100; Visual Sonics, Toronto, ON, Canada), as described (Urboniene et al., 2010; Tian et al., 2017). The following variables were measured: pulmonary artery acceleration time (PAAT), main pulmonary artery (PA) inner diameter at the level of the pulmonary outflow tract during mid-systole, diastolic and systolic thickness of the RV free wall (RVFW), and tricuspid annular plane systolic excursion (TAPSE). RVFW systolic thickening was calculated as (RVFW_systole_ – RVFW_diastole_)/RVFW_diastole_, and cardiac output (CO) was estimated as HR × VTI × ID^2^/4, where HR is the heart rate, VTI is the systolic velocity time integral over the main PA flow obtained from pulsed-wave Doppler, and ID is the inner diameter of the main PA at mid-systole, as described previously (Piao et al., 2010; Urboniene et al., 2010; Prins et al., 2017; Tian et al., 2017).

### Right Heart Catheterization (RHC)

At week 4, following cardiac ultrasound, invasive closed-chest RHC was performed to obtain RV pressure-volume loops. Briefly, rats were anesthetized with 5% isoflurane induction and maintained with 3% during procedures. A high-fidelity catheter (Scisense pressure-volume catheter; Transonic, London, ON, Canada) was advanced to RV through the right jugular vein and the right atria in closed-chest animals. During catheterization, animals were intubated and ventilated. RV pressure and volume were recorded continuously using Scisense ADV500 Pressure-Volume Measurement System (Transonic, London, ON, Canada) and LabScribe2 software (iWorx, Dover, NH, United States). RV systolic pressure and end-diastolic pressure (RVSP and RVEDP, respectively) were directly obtained from the pressure trace. Total pulmonary resistance (TPR) was then calculated as mPAP/CO, where CO is cardiac output calculated as (RV end-diastolic volume - RV end-systolic volume) × heart rate, and mPAP is the mean pulmonary artery pressure estimated as 0.61 × RVSP + 2 (Chemla et al., 2004).

### Histological Analysis

After RHC, animals were sacrificed. RV and LV plus septum were then dissected for tissue weight measurement. Biopsies of RV free wall tissues were fixed in 10% buffered formalin. The fixed tissues were then embedded in paraffin and stained with picrosirius red, for measurement of collagen deposition.

Images of RV stained with picrosirius red were taken by a scientist who was blinded to the experimental groups, using a Leica digital color camera (Leica DFC310 FX, Leica Microsystems; Wetzlar, Germany) and Leica DM4000 B LED microscope with a 20X objective (Leica Microsystems; Wetzlar, Germany). For each sample, more than 4 areas were imaged and analyzed for the percentage of the collagen area using Leica software (LAS V4.7, Leica Microsystems; Wetzlar, Germany). Results are presented as the average percentage of all the sample areas stained with picrosirius red.

### RV Fibroblasts Isolation

RVfib were isolated from control and monocrotaline rats (*n* = 8 each) using a modification of a previously described method (Agocha and Eghbali-Webb, 1997). Briefly, excised fresh RV free wall tissues were rinsed with ice-cold PBS twice and minced in ice-cold PBS into small pieces at ∼0.5 mm. The minced tissues were then digested at 37°C in 2 mL Hanks’ Balanced Salt Solution supplemented with 0.1% trypsin-EDTA and 200 U/mL collagenase in 15 mL conical tube by constant stirring at a speed of 1000 RPM via Eppendorf^TM^ ThermoMixer temperature control device (05412503; Thermo Fisher Scientific, Waltham, MA, United States) for 5 min, and the supernatant was discarded. A total of 2 mL digestion solution was added to the 15 mL conical tube for the next digestion. The second to the sixth digestions underwent constant stirring for 15 min each, and at the end of each digestion period the supernatant was aspirated and centrifuged. The pellet was suspended in culture medium and placed on a 100-mm culture dish (12-556-02; Fisher Scientific, Waltham, MA, United States). After the supernatant was aspirated, 2 mL of fresh digestion solution was added for the next cycle. On the second and the third days after the cell isolation, the cell culture dish was washed with PBS and replaced with new culture medium. Starting from the fourth day, the culture medium was replaced every 2 or 3 days. The identity of fibroblasts was confirmed by immunofluorescence using previously published criteria (positive for vimentin and negative for α-smooth muscle actin, von Willebrand factor, and heavy chain cardiac myosin) (Neuss et al., 1996; Agocha and Eghbali-Webb, 1997).

### Cell Culture

Isolated RVfib were cultured in DMEM containing glucose (4500 mg/L), supplemented with 2 mM L-glutamine, 10% fetal bovine serum, penicillin/streptomycin (100 U/mL), and 4.6 ng/L fibroblast growth factor basic. Cells were treated with P110 or control peptide TAT (10, 50, 100 μM or 1 mM) daily, with small interfering RNA of Drp1 (siDrp1) or its negative control (NC1), or with Mdivi-1 (25 μM) or DMSO once. Depending on the experiment they were studied 5, 24, 48, or 72 h post incubation with the study drug.

### qRT-PCR

The mRNA was extracted from RVfib using the Invitrogen^TM^ Ambion PureLink RNA Mini Kit (12183025; Thermo Fisher Scientific, Waltham, MA, United States), and then converted to cDNA with High-Capacity cDNA Reverse Transcription Kit (4368814; Thermo Fisher Scientific, Waltham, MA, United States). mRNA levels of Drp1, Fis1, MFF, MiD49, and MiD51 were assessed by Bio-Rad CFX96 qPCR instrument (Mississauga, ON, Canada). mRNA expression was normalized to GAPDH mRNA and the relative expression between groups was assessed using 2^-ΔΔCt^ equation. All the primers were purchased from IDT (San Jose, CA, United States).

#### Mitochondrial Networking

Mitochondrial fragmentation was evaluated with MitoTracker^TM^ Green FM (M7514; Thermo Fisher Scientific, Waltham, MA, United States) or tetramethylrhodamine methyl ester (TMRM; Cat #T668, Lifetechnologies; Carlsbad, CA, United States). Briefly, RVfib were cultured in a 35-mm glass-bottom dish (P35G-1.5-14-C; MatTek Corporation, Ashland, MA, United States) and incubated in culture medium containing 400 nM MitoTracker^TM^ Green FM or 25 nM TMRM at 37°C in the dark for 40 or 20 min, respectively. Images were then taken with a Leica SP8 confocal, laser-scanning microscope (Leica Microsystems; Wetzlar, Germany) with a 1.40 NA, 63X oil immersion objective with 3X digital zoom. Mitochondrial segments were identified using ImageJ (National Institutes of Health, Bethesda, MD, United States), and mitochondrial fragmentation count was calculated as the ratio of the number of individual mitochondria and the total area of these mitochondria, as described (Marsboom et al., 2012; Rehman et al., 2012).

In addition, mitochondrial morphology was also quantified via a machine-learning algorithm using the Leica LAS X software (Leica Microsystems; Wetzlar, Germany), as described (Chen et al., 2018). This algorithm automatically measures the percentage area of three morphology-based categories (punctate, intermediate, and filamentous) in an entire field of cells, independent of the operator. Briefly, an image that has mitochondria covering a large range of area, length, and aspect ratio was chosen, and more than 15 mitochondria from each category were manually selected from this image to inform the machine-leaning algorithm. Subsequently, the algorithm was applied to all the images to obtain the percentage area of each category in each image. Results are presented as the average distribution of the three categories.

#### Proliferation Assay

Cell proliferation was assessed using the Click-iT^®^ EdU Flow Cytometry Assay Kit following the manufacturer’s instructions (C10420; Thermo Fisher Scientific, Waltham, MA, United States), as described (Hong et al., 2017). Briefly, 10 μM EdU (5-ethynyl-2′-deoxyuridine) was added to the culture medium for incorporation into DNA during active DNA synthesis and the cell culture dish that did not have addition of EdU was taken as a negative staining control. A total of 24 h following the addition of EdU, cells were harvested, fixed, permeabilized, and labeled with Alexa Fluor^®^ 488 azide. The analysis for proliferation was then performed using the analysis mode of the flow sorter SH800S (Sony Biotechnology Inc., San Jose, CA, United States). A total of 10,000 events were recorded, adjusted according forward and side scatter and the positive population was gated and analyzed using the 488 nm laser and FL2 filter (525 ± 50).

### Immunoblotting

Proteins were extracted from RVfib with cell lysis buffer (#9803; Cell Signaling Technologies, Beverly, MA, United States) and 50 or 70 μg (for phosphorylated Drp1 at Serine 616) protein was loaded to SDS-PAGE gel for immunoblotting. Images were taken with Chemidoc MP Imaging System (Bio-Rad Laboratories; Mississauga, ON, Canada) and analyzed with ImageJ (National Institutes of Health, Bethesda, MD, United States). The following antibodies were used: anti-β-actin (A5441; Sigma, St. Louis, MO, United States), anti-MFN2 (ab56889; Abcam, Cambridge, MA, United States), anti-phospho-Drp1 (Ser616) (#3455; Cell Signaling Technology, Danvers, MA, United States), anti-Drp1 (611112; BD Transduction Laboratories^TM^, San Jose, CA, United States). β-actin was used as loading control. Note that for the gels running for phosphorylated Drp1 at Serine 616, the cells were collected on ice and low temperature was carefully maintained throughout all steps of protein isolation and processing.

### Immunofluorescence

Cells were fixed in 4% paraformaldehyde at room temperature for 10 min, permeabilized with 1% Triton X-100 in PBS, blocked in 2% bovine serum albumin plus 0.05% Tween-20 in PBS for 30 min, and incubated with primary antibodies at 4°C overnight. The following primary antibodies were used: anti-collagen III (ab6310; Abcam, Cambridge, MA, United States), anti-collagen I (ab34710; Abcam, Cambridge, MA, United States), anti-phospho-Drp1 (Ser616) (#3455; Cell Signaling Technology, Danvers, MA, United States), anti-vimentin (ab8069; Abcam, Cambridge, MA, United States), anti-α-smooth muscle actin (ab5694; Abcam, Cambridge, MA, United States), anti-von Willebrand factor (ab6994; Abcam, Cambridge, MA, United States), and anti-heavy chain cardiac myosin (ab15; Abcam, Cambridge, MA, United States). Slides were then washed with PBS for 5 min three times and then incubated with secondary antibodies (Alexa Fluor-conjugated secondary antibodies Alexa Fluor 488 goat anti-rabbit #A-11034 and Alexa Fluor goat anti-mouse #A-11031; Invitrogen, Carlsbad, CA, United States) for 60 min at room temperature. Finally, slides were washed with PBS three times in the dark and mounted in ProLong^TM^ Gold Antifade Mountant with DAPI (P36935; Thermo Fisher Scientific, Waltham, MA, United States). Images on phospho-Drp1 (Ser616) and collagen were taken with a Leica SP8 confocal, laser-scanning microscope (Leica Microsystems; Wetzlar, Germany) with a 1.40 NA, 63X oil immersion objective with 3X digital zoom and with Leica DM4000 B LED microscope with a 20X objective (Leica Microsystems; Wetzlar, Germany). A microscopist blinded to treatment groups performed the analysis. Images for cell characterization were acquired with an EVOS image system (EVOS FL Color, Life Technologies; Carlsbad, CA, United States). The intensity of fluorescent signal for phospho-Drp1 (Ser616) and collagen I and III was measured using ImageJ software (National Institutes of Health; Bethesda, MD, United States).

### Statistical Analysis

All of the data are reported as mean ± standard error of the mean (SEM). Two-tailed, Student’s *t*-test, paired *t*-test, Chi-Square test, or analysis of variance (ANOVA) was performed as appropriate. Statistical analyses were performed using the GraphPad Prism version 7.04 for Windows (GraphPad^1^ Software, La Jolla, CA, United States). A *P* < 0.05 was considered statistically significant.

## Results

### Mdivi-1, siDrp1, and P110 at High Dose Inhibit Mitochondrial Fission in MCT-RV Fibroblasts

**FIGURE 1:**
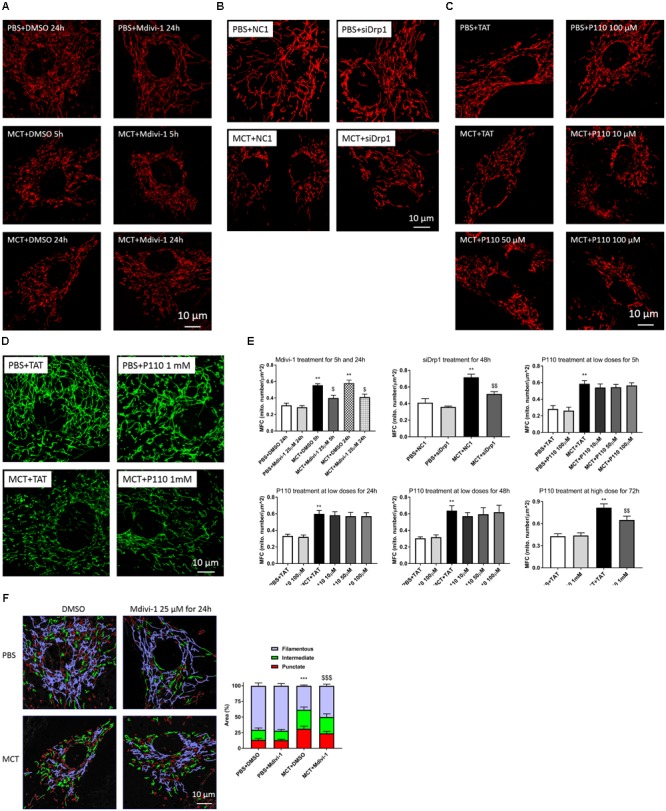
Monocrotaline (MCT)-induced RVfib display mitochondrial fragmentation and mitochondrial division inhibitor 1 (Mdivi-1; 25 μM) for both 5 and 24 h, small interfering RNA targeting Drp1 (siDrp1) for 48 h, and P110 treatment at high dose (1 mM) for 3 days inhibited mitochondrial fission, whereas P110 at a dose up to 100 μM for 5, 24, and 48 h has no significant effect. Representative mitochondrial network stained with TMRM in RVfib with and without **(A)** Mdivi-1 for 5 and 24 h, **(B)** siDrp1 for 48 h, or **(C)** P110 (up to 100 μM) treatment for 5 h; **(D)** Representative mitochondrial network stained with MitoTracker^TM^ Green FM with P110 and its control TAT (1 mM) treatment for 3 days; **(E)** Summary of mitochondrial fragmentation count (MFC) in RVfib; **(F)** Representative mitochondrial network divided into three categories (punctate, red; intermediate, green; filamentous, purple) in RVfib treated with Mdivi-1 (25 μM) for 24 h and summary of area distribution of the three categories. ^∗∗^*P* < 0.01 and ^∗∗∗^*P* < 0.001 versus PBS+DMSO, PBS+NC1, or PBS+TAT group; ^$^*P* < 0.05, ^$$^*P* < 0.01, and ^$$$^*P* < 0.001 versus the corresponding vehicle control group (i.e., MCT+DMSO, MCT+NC1, or MCT+TAT). *n* = 5 per group except for the PBS+NC1 and PBS+siDrp1 groups (*n* = 2).

The identity of isolated fibroblasts from RV was confirmed with immunofluorescence positive for vimentin and negative for α-smooth muscle actin, von Willebrand factor, and heavy chain cardiac myosin staining (Supplementary Figure S1). Compared to control-RVfib, MCT-RVfib displayed excessive mitochondrial fission indicated by increased mitochondrial fragmentation count (MFC) and decreased percentage area of filamentous mitochondria (**Figure 1**). Both Mdivi-1 (25 μM) treatment for 5 or 24 h and siDrp1 for 48 h inhibited mitochondrial fission in MCT-RVfib, whereas P110 treatment for either 5, 24, or 48 h at a dose of 10, 50, or 100 μM did not significantly inhibited mitochondrial fission in MCT-RVfib (**Figure 1** and Supplementary Figure S2). Treatment with P110 at a high dose (1 mM) for 3 days significantly inhibited mitochondrial fission in MCT-RVfib with no effect on Control-RVfib (**Figures 1D,E**).

### Mdivi-1 and P110 at High Dose Reduce Proliferation in MCT-RV Fibroblasts

**FIGURE 2:**
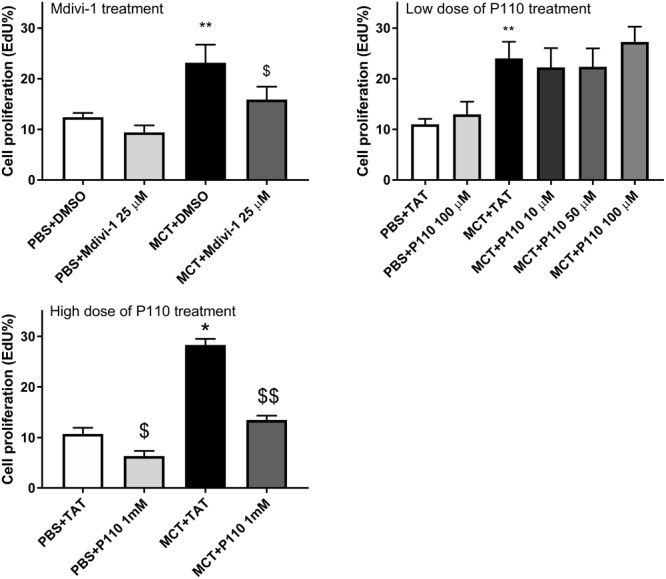
Compared to control, monocrotaline (MCT)-induced RVfib are hyperproliferative as measured by Click-iT^®^ EdU assay. Mitochondrial division inhibitor 1 (Mdivi-1; 25 μM) for 2 days and high dose of P110 (1 mM) for 3 days reduced proliferation in MCT-RVfib whereas lower doses of P110 had no effect. ^∗^*P* < 0.05 and ^∗∗^*P* < 0.01 versus PBS+DMSO or PBS+TAT group; ^$^*P* < 0.05 and ^$$^*P* < 0.01 versus the corresponding vehicle control group. *n* = 5 per group.

Compared to Control-RVfib, MCT-RVfib had higher proliferation rates (**Figure 2**). Mdivi-1 (25 μM) treatment for 48 h significantly reduced proliferation rates in MCT-RVfib (**Figure 2**). Treatment with P110 for 48 h at a dose of 10, 50, or 100 μM had no significant effect on the proliferation of MCT-RVfib (**Figure 2**). Again, treatment with P110 at a high dose (1 mM) for 3 days significantly reduced proliferation rates in MCT-RVfib but also in Control-RVfib (**Figure 2**). Preliminary study found that treatment with P110 at low doses (0.5 mM or lower) for 3 days did not reduce proliferation rates on RV fibroblasts from two MCT rats (data not shown).

### Mdivi-1 Reduces Collagen Production in MCT-RV Fibroblasts

**FIGURE 3:**
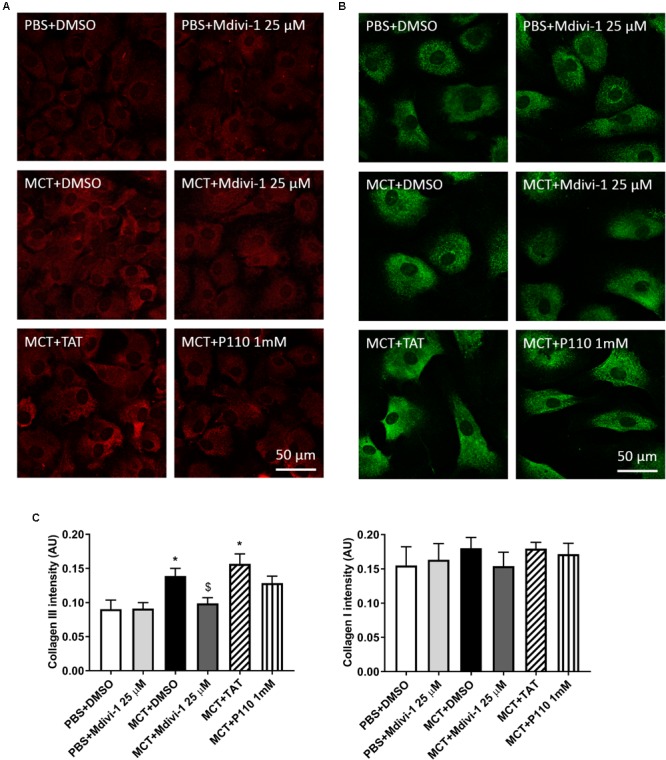
Compared to control, monocrotaline (MCT)-induced RVfib have significantly greater production of collagen type III but not collagen type I. Mitochondrial division inhibitor 1 (Mdivi-1; 25 μM) significantly reduced type III collagen production and P110 (1 mM) for 3 days showed a trend in reducing type III collagen production (*P* = 0.14). Representatives of **(A)** type III (red) and **(B)** type I (green) collagen in RVfib characterized by immunofluorescence; **(C)** Summary of intensity of the fluorescent signal. ^∗^*P* < 0.05 versus PBS+DMSO group; ^$^*P* < 0.05 versus MCT+DMSO group. *n* = 5∼6 per group.

Compared to Control-RVfib, MCT-RVfib increased collagen production in both types I and III, though only statistically significantly in type III (**Figure 3**). Mdivi-1 (25 μM) for 3 days significantly reduced type III collagen production in MCT-RVfib, while P110 (1 mM) for 3 days showed a trend (*P* = 0.14) in reducing type III collagen production in MCT-RVfib (**Figure 3**).

### MCT-RV Fibroblasts Have Increased Phosphorylated Drp1 at Serine 616

**FIGURE 4:**
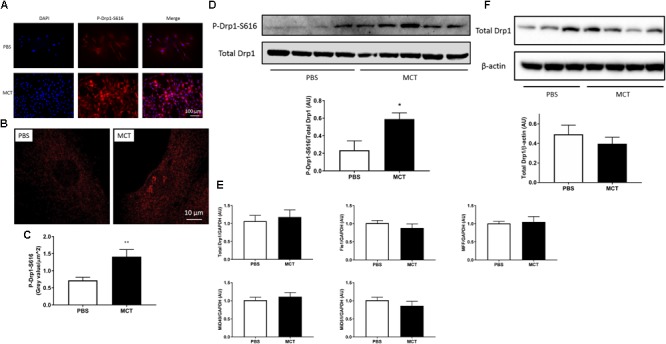
Compared to control, monocrotaline (MCT)-induced RVfib have upregulation of phosphorylation of dynamin-related protein 1(Drp1) at Serine 616 but no significant changes in total Drp1 or Drp1 binding partners. Representative immunofluorescence images of phosphorylated Drp1 at Serine 616 (P-Drp1-S616; red) in RVfib **(A)** at 20X with DAPI at blue and **(B)** at 63X; **(C)** Summary of the intensity of phosphorylated Drp1 at Serine 616 (*n* = 5∼6 per group); **(D)** Immunoblotting on P-Drp1-S616 and its ratio to total Drp1 (*n* = 4∼5 per group); **(E)** mRNA expression of total Drp1, fission protein 1 (Fis1), mitochondrial fission factor (MFF), and mitochondrial dynamics proteins of 49 and 51 kDa (MiD49 and MiD51, respectively) in RVfib (*n* = 6∼8 per group); **(F)** Immunoblotting on total Drp1 in RVfib (*n* = 4∼6 per group). ^∗^*P* < 0.05 and ^∗∗^*P* < 0.01 versus PBS group.

Activated Drp1 (phosphorylation at Serine 616) measured via immunofluorescence was significantly increased in MCT-RVfib versus Control-RVfib (**Figures 4A–C**), which is confirmed with immunoblotting (**Figure 4D**). qRT-PCR measurement showed that there was no significant difference between Control-RVfib and MCT-RVfib in the mRNA expression of total Drp1, Fis1, MFF, MiD49, or MiD51 (**Figure 4E**). Immunoblotting also did not find significant change in total Drp1 in MCT-RVfib versus the control (**Figure 4F**). In addition, qRT-PCR measurement found no significant difference between groups in the mRNA expression of fusion mediators (MFN1, MFN2 and OPA1) (Supplementary Figure S3). However, there was a trend toward reduction in the expression of MFN2 mRNA (*P* = 0.32) and protein (*P* = 0.18) in MCT-RVfib versus Control-RVfib (Supplementary Figures S3, S4).

### P110 Does Not Improve RV Function in MCT Rats

**FIGURE 5:**
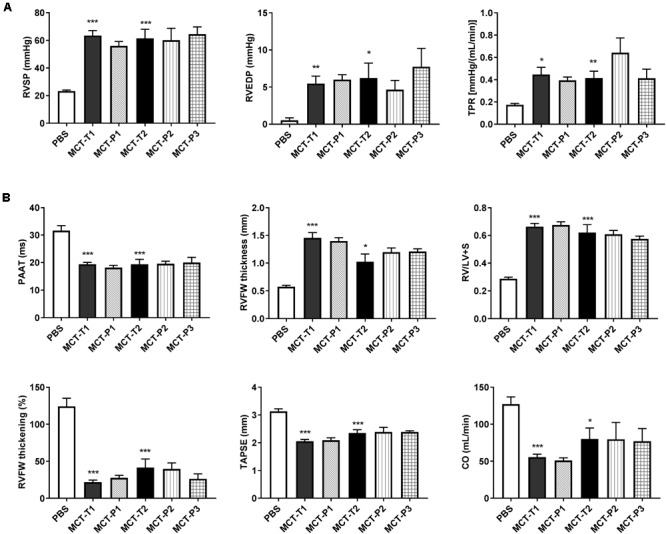
Monocrotaline (MCT) rats developed RV hypertrophy and pulmonary hypertension and P110 did not improve RV function. **(A)** RV systolic and diastolic pressures (RVSP and RVEDP) and total pulmonary resistance (TPR) increased in MCT rats and were not changed by P110 treatment; **(B)** MCT decreased pulmonary artery acceleration time (PAAT) and caused increases in RV free wall (RVFW) thickness and the ratio of RV over LV plus septum weight (RV/LV+S). MCT rats had reduced RVFW thickening, tricuspid annular plane systolic excursion (TAPSE), and cardiac output (CO). Treatment of P110 did not change any of these parameters in MCT rats. MCT-T1 and MCT-P1, MCT rats treated with TAT and P110, respectively, on day 14 and 19; MCT-T2 and MCT-P2, MCT rats treated with TAT and P110, respectively, every other day starting from day 10; MCT-P3, MCT rats treated with P110 only once immediately before the injection of MCT. ^∗^*P* < 0.05, ^∗∗^*P* < 0.01, and ^∗∗∗^*P* < 0.001 versus PBS group. *n* = 4∼10 per group.

At week 4 post-injection of MCT or PBS, the body weight of MCT rats was significantly less than PBS (i.e., control) rats and was not altered by P110 treatment (either prevention or regression) (Supplementary Figure S5). Compared to control (i.e., PBS), MCT rats had significantly increased total pulmonary resistance (TPR, estimated from RHC; **Figure 5A**) and developed RV hypertrophy (RVH) (**Figure 5B**). MCT rats had higher pressure in both PA and RV as indicated by shorter pulmonary artery acceleration time (PAAT) (measured by echocardiography; **Figure 5B**) and higher RV systolic pressure (RVSP) and RV end-diastolic pressure (RVEDP) (measured by RHC; **Figure 5A**). Also, MCT rats had reduced RV contractility manifest as reduced RVFW systolic thickening, reduced tricuspid annular plane systolic excursion (TAPSE), and reduced cardiac output (CO) (**Figure 5B**). P110 treatment did not improve RV function in MCT rats in either the prevention protocol (P110 was administered at the time of MCT injection) or the two regression protocols (P110 administered on day 14 and 19 or every other day from day 10) (**Figure 5**).

### MCT RV Develops Fibrosis That Is Not Altered by P110 Treatment

**FIGURE 6:**
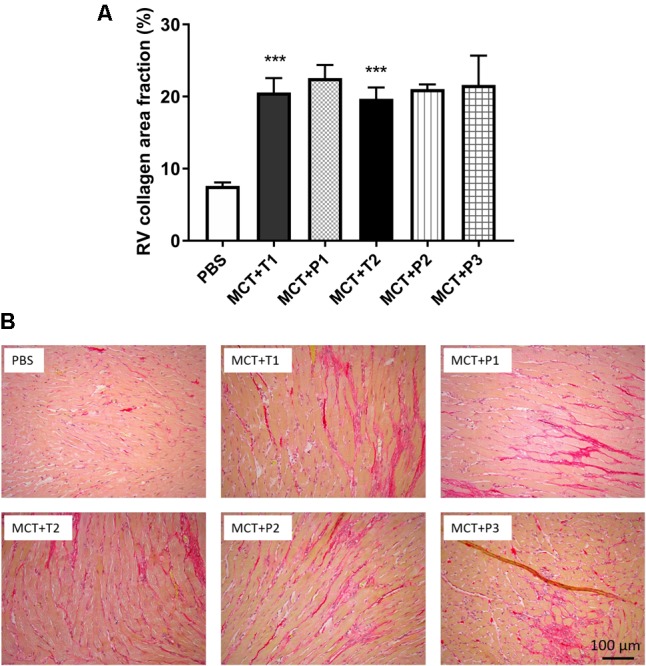
Collagen deposition in RV is increased monocrotaline (MCT) rats and not changed by P110 treatment. **(A)** Representative images of RV stained with picrosirius red for collagen and **(B)** summary of collagen area fraction. MCT-T1 and MCT-P1, MCT rats treated with TAT and P110, respectively, on day 14 and 19; MCT-T2 and MCT-P2, MCT rats treated with TAT and P110, respectively, every other day starting from day 10; MCT-P3, MCT rats treated with P110 only once immediately before the injection of MCT. ^∗∗∗^*P* < 0.001 versus PBS group. *n* = 4∼10 per group.

Compared to control, MCT rats developed greater RV fibrosis, as observed from picrosirius red staining (**Figure 6**). *In vivo* P110 treatment did not prevent or regress RV fibrosis in MCT rats (**Figure 6**).

## Discussion

This study examined the role of disordered mitochondrial dynamics in the hyperproliferative, collagen-producing phenotype of RV fibroblasts derived from rats with MCT-induced PAH. The study revealed five significant findings. First, MCT-RVfib display excessive mitochondrial fission and this phenotype persists in culture. Second, MCT-RVfib are hyperproliferative. Third, MCT-RVfib have increased expression of activated Drp1 (phosphorylated at Serine 616). Fourth, the mitochondrial fission is crucial to the hyperproliferative, profibrotic phenotype, since Mdivi-1 treatment, which inhibits mitochondrial fission, also reduces proliferation and collagen production, indicating Drp1 is a potential antifibrotic target. Fifth, P110, a competitive peptide that antagonizes the interaction between Drp1 and Fis1 also reduces mitochondrial fission and RVfib proliferation *in vitro*; however, it only has these effects at very high doses. Moreover, P110 failed to prevent or regress RV fibrosis or improve RV function *in vivo*. These data suggest that Drp1-mediated fission is central to the RVfib hyperproliferative, profibrotic phenotype in MCT-PAH.

Changes in mitochondrial dynamics regulates vital cellular functions including metabolism, cell cycle progression, and apoptosis (Archer et al., 2008; Suen et al., 2008; Rehman et al., 2012). Excessive mitochondrial fission has been observed in PAH in RV myocytes (Tian et al., 2017), pulmonary artery smooth muscle cells (PASMC) (Marsboom et al., 2012; Chen et al., 2018), and pulmonary adventitial fibroblasts (Plecita-Hlavata et al., 2016). In PASMC, excessive mitotic fission is associated with increased proliferation and is thought to reflect coordination between division of the nucleus and mitochondria (Marsboom et al., 2012; Chen et al., 2018). In PAH PASMC, this fissogenic phenotype is mediated by both posttranslational modification of Drp1, leading to its activation, and increased interaction of Drp1 and upregulation of its recently discovered binding partners, MiD49 and MiD51 (Chen et al., 2018). Similar to PAH PASMC, we also found that RVfib in MCT-PAH display more mitochondrial fragmentation (**Figure 1**) and increased proliferation (**Figure 2**), confirming the persistence of a phenotype characterized by excess fission and rates of fibroblast proliferation in culture. To the best of our knowledge, this is the first study to demonstrate mitochondrial fission and show that it regulates the proliferation and collagen production of in RVfib in PAH, or indeed in any cardiac disease. We speculate that epigenetic mechanisms, triggered by MCT, are in play, since the phenotype persists through multiple passages of fibroblasts in culture of these cells, which are derived from genetically normal rodents.

Mechanistically, the mitochondrial network is regulated by fission mediator (Drp1) and fusion mediators (MFN1, MFN2, OPA1). Increases in Drp1 or decreases in fusion mediators result in mitochondrial fragmentation in both PASMC and pulmonary artery adventitial fibroblasts in PAH (Marsboom et al., 2012; Ryan et al., 2013; Plecita-Hlavata et al., 2016; Chen et al., 2018). Our group has previously found an increase in the phosphorylated Drp1 at Serine 616 and a decrease in MFN2 in PASMC in PAH (Marsboom et al., 2012; Ryan et al., 2013). Studies on pulmonary artery adventitial fibroblasts from the lungs in PAH found a decrease in both MFN2 and OPA1 (Plecita-Hlavata et al., 2016). Consistent with this we found an increase in the phosphorylated Drp1 at Serine 616 (**Figures 4A–D**) and a strong trend to decreased MFN2 expression (Supplementary Figures S3, S4) in RVfib in MCT-PAH, without significant changes in the other two fusion mediators (MFN1 and OPA1) (Supplementary Figure S3). Therefore, our data suggest that acquired activation of Drp1 promotes mitochondrial fragmentation in MCT-RVfib, perhaps reinforced by reduced expression of the fusion mediator MFN2.

Since Drp1 mediates mitochondrial mitotic fission, which is associated with accelerated cell cycle progression and increased cell proliferation, Drp1 has been proposed as a therapeutic target for PAH (Marsboom et al., 2012). Mdivi-1, which inhibits a conformational change of Drp1 required for self-assembly and GTP hydrolysis, is a selective Drp1 GTPase activity inhibitor (Cassidy-Stone et al., 2008). Inhibiting Drp1 via Mdivi-1 inhibits mitochondrial fission and reduces proliferation in PASMC in PAH (Marsboom et al., 2012). In agreement with the study on PASMC, the current study demonstrates the same effects of Mdivi-1 on RVfib in MCT-PAH *in vitro*, i.e., the inhibition of mitochondrial fission and reduction in proliferation (**Figures 1, 2**). The inhibition of mitochondrial fission is also achieved by knocking down Drp1 (i.e., siDrp1; **Figure 1**), providing additional molecular certainty that it is Drp1 which is crucial to the fragmented mitochondrial morphology in MCT-RVfib.

Along with excessive mitochondrial fission and hyperproliferation, MCT-RVfib also have greater collagen production than Control-RVfib (**Figure 3**), consistent with the increased RV fibrosis observed in MCT versus control rats (**Figure 6**). Mdivi-1 also reduced collagen type III production in MCT-RVfib (**Figure 3**), indicating that mitochondrial fission is linked to collagen production and that Drp1 is a potential antifibrotic target.

Activated Drp1 translocates to the mitochondrion where it associates with its binding partners creating a ring-like, multimeric structure which constricts and divides the mitochondrion. The relevant binding partner varies by cell type. For example, in cardiomyocytes, Drp1 binds to Fis1 but not MiD51 or MFF under conditions of acute ischemia-reperfusion injury (Disatnik et al., 2013). In the MCT RV, P110 (1 μM) both inhibits mitochondrial fission and improves RV myocyte and cardiac diastolic function in ischemia-reperfusion injury (Tian et al., 2017). In contrast, in the current study, only 1000-fold higher doses of P110 were able to inhibit mitochondrial fission or reduce proliferation in MCT-RVfib *in vitro* (**Figures 1, 2**). At these doses, the specificity of P110, a competitive peptide which theoretically only inhibits the interaction between Fis1 and Drp1, is unknown. This basis for the difference between the cardiomyocytes and RVfib studies is unknown. *In vivo* P110 failed to prevent or regress RV fibrosis. Our protocol involved repeated administration of P110 over 2 weeks. In contrast, Disatnik et al. (2013) gave P110 treatment immediately prior to a single point injury (acute ischemia-reperfusion injury) and observed a beneficial effect. We speculate that MCT, which causes a complex and sustained injury, may be less amenable to intervention (**Figures 5, 6**). Previous studies have used a high dose of P110 at 3 mg/kg/day using Alzet osmotic mini-pumps on mouse models of Huntington’s disease and amyotrophic lateral sclerosis and shown benefits of P110 (Guo et al., 2013; Joshi et al., 2018). In addition, the half-life of P110 is probably as short as 1 h (Qi et al., 2013), perhaps higher doses of P110 and use of continuous infusion protocols might have proven more effective. Alternatively, Fis1 may simply be less relevant as a Drp1 binding partner in MCT-RVfib than other binding partners, which we did not assess (MFF, MiD49, and MiD51).

Nonetheless, the fact that P110 at high dose also inhibited mitochondrial fission and cell proliferation (**Figures 1, 2**) may indicate a role for Drp1–Fis1 interaction, as observed in the RV myocytes in this MCT model of PAH (Tian et al., 2017). However, we were only able to observe an effect of P110 at 1 mM dose and the effect required several days of incubation. This may indicate that the observed effects are nonspecific or that the interaction between Drp1 and Fis1 in RV fibroblasts is robust and hard to reverse. Certainly, at the doses of P110 that we could afford to test we could neither prevent nor regress RV fibrosis *in vivo* (**Figure 6**). This would favor the interpretation that the Drp1–Fis1 interaction may be less critical in RVfib than in RV myocytes.

The finding that P110 treatment did not change PAAT or pulmonary vascular resistance (or TPR) in MCT rats (**Figure 5**) indicates Drp1–Fis1 interaction is not important either for pulmonary vasculature at least in MCT-PAH. This confirms a recent study from our group. Although Fis1 is found to be upregulated in PASMC in human PAH patients (Marsboom et al., 2012; Ryan et al., 2013), knockdown of Fis1 using a small interfering RNA (siRNA) does not inhibit mitochondrial fission nor reduce proliferation in PASMC in human PAH patients (Chen et al., 2018).

Drp1 may use different binding partners to facilitate mitochondrial fission in RVfib in PAH. We recently showed that two Drp1 binding partners (MiD49 and MiD51) are upregulated in PASMC in PAH. The epigenetic upregulation of MiDs (mediated by a reduced expression of microRNA 34a-3p) promotes a Drp1-dependent increase in mitochondrial fission and proliferation in PASMC in both human PAH patients and MCT rats (Chen et al., 2018). In this study, mRNA expression of MiD49 and MiD51 was not changed in MCT-RVfib (**Figure 4E**), although mRNA and protein expression levels are not always concordant. The role of MiDs on mitochondrial fission in RVfib remains unknown.

### Limitations

Several limitations are acknowledged. First, we did not examine if Mdivi-1 can reduce RV fibrosis in MCT rats. Our group has previously shown that Mdivi-1 can improve RV function in MCT rats (Marsboom et al., 2012). In addition, another study demonstrates that Mdivi-1 can reduce LV fibrosis in aortic banding model in rats (Givvimani et al., 2012). We propose (but did not prove) that Mdivi-1 would reduce RV fibrosis in PAH. This requires direct confirmation in a future *in vivo* study.

Second, we did not measure the expression of Drp1 phosphorylated at Serine 637. Decreased phosphorylation of Drp1 at Serine 637 contributes to ischemia-reperfusion injury in cardiac arrest (Cereghetti et al., 2008; Chang and Blackstone, 2010; Sharp et al., 2014, 2015). In future studies, measurement of this phosphorylation of Drp1 in RVfib will be important to better determine the mechanism of Drp1 activation.

Third, we did not perform immunoprecipitation studies on RVfib to directly examine the role of Drp1–Fis1 interaction in MCT-RVfib. Indeed, previous *in vitro* studies (Disatnik et al., 2013) showed that P110 at 1 μM (which is a low dose) inhibited ischemia-induced mitochondrial fission in cardiac myocytes. In ischemia-reperfusion injury, the Drp1–Fis1 interaction is well established as an early step in the generation of the mitochondrial-derived reactive oxygen species that drives cardiac dysfunction. In contrast, in RV fibroblasts, P110 at this dose (1 μM) had no effect. This may be due to the difference in sensitivity of the Drp1–Fis1 interaction in different cell types (cardiomyocytes versus cardiac fibroblasts) and/or a different role for Fis1 in different pathologic situations (more important in acute ischemia than in the MCT model of chronic, pressure-volume overload). The reason why such high doses of P110 were required to have significant effects on RVfib *in vitro* is unclear. In the future, immunoprecipitation studies will be used to clarify the role of high dose P110 on Drp1–Fis1 interaction.

Fourth, because P110 only blocks the interaction between Drp1 and Fis1, the failure of P110 treatment *in vivo* may indicate either that adequate levels of P110 were not achieved *in vivo* and/or that the Drp1–Fis1 interaction is less important in the pathogenesis of the fragmented mitochondria-proliferative fibroblast phenotype than other Drp1-binding partner interactions (such as Drp1-MiD49 and Drp1-MiD51). The role of MiD49 and MiD51 in MCT-RVfib remains unknown and requires further study.

## Conclusion

We conclude that in MCT-induced PAH, RV fibroblasts display mitochondrial fragmentation that reflects Drp1-mediated fission. Increased mitochondrial fission promotes a hyperproliferative state and results in excessive production of collagen type III. Inhibiting Drp1 can inhibit mitochondrial fission, and reduce fibroblast proliferation and collagen production, suggesting Drp1 is a potential antifibrotic target. Further *in vivo* preclinical studies are required to establish the translational relevance of these observations.

## Author Contributions

LT and SA designed the work. LT, FP, DW, AD, K-HC, JM, and PL performed the experiments. LT, FP, DW, AD, K-HC, JM, PL, and SA analyzed and interpreted the data. LT drafted the work. LT, FP, DW, AD, K-HC, JM, PL, and SA revised the work critically and performed the final approval of the version to be published.

## Conflict of Interest Statement

The authors declare that the research was conducted in the absence of any commercial or financial relationships that could be construed as a potential conflict of interest.

## Footnotes

1 www.graphpad.com
