# Supplementary material for: Increased Drp1-Mediated Mitochondrial Fission Promotes Proliferation and Collagen Production by Right Ventricular Fibroblasts in Experimental Pulmonary Arterial Hypertension
Source: Front Physiol. 2018 Jul 10;9:828. doi: 10.3389/fphys.2018.00828 (PMC6048272; doi:10.3389/fphys.2018.00828)

Frontiers in Physiology, 2018
Electronic Supplemental Material

Increased Drp1-mediated mitochondrial fission promotes proliferation and collagen production by right ventricular fibroblasts in experimental pulmonary arterial hypertension

Lian Tian, Francois Potus, Danchen Wu, Asish Dasgupta, Kuang-Hueih Chen, Jeffrey Mewburn, Patricia Lima, Stephen L. Archer*

Department of Medicine, Queen’s University, Kingston, Ontario, Canada

* Corresponding author
Stephen L. Archer MD, FRCP(C), FAHA, FACC
Tier 1 CRC Mitochondrial Dynamics
Professor, Head Department of Medicine, Queen's University
Program Medical Director Kingston General Hospital (KGH), Hotel Dieu Hospital (HDH),
and St. Mary’s of the Lake Hospital (SMOL)
Queen’s Cardiopulmonary Unit (QCPU), Translational Institute of Medicine (TIME)

Etherington Hall, Room 3041
94 Stuart St., Kingston, Ontario, Canada, K7L 3N6
Preferred E-mail: stephen.archer@queensu.ca

Supplemental Figure S1. Confirmation of the identity of isolated RV fibroblasts via immunofluorescence. Staining is positive for vimentin (red) and negative for α-smooth muscle actin, von Willebrand factor and heavy chain cardiac myosin. DAPI is in blue.

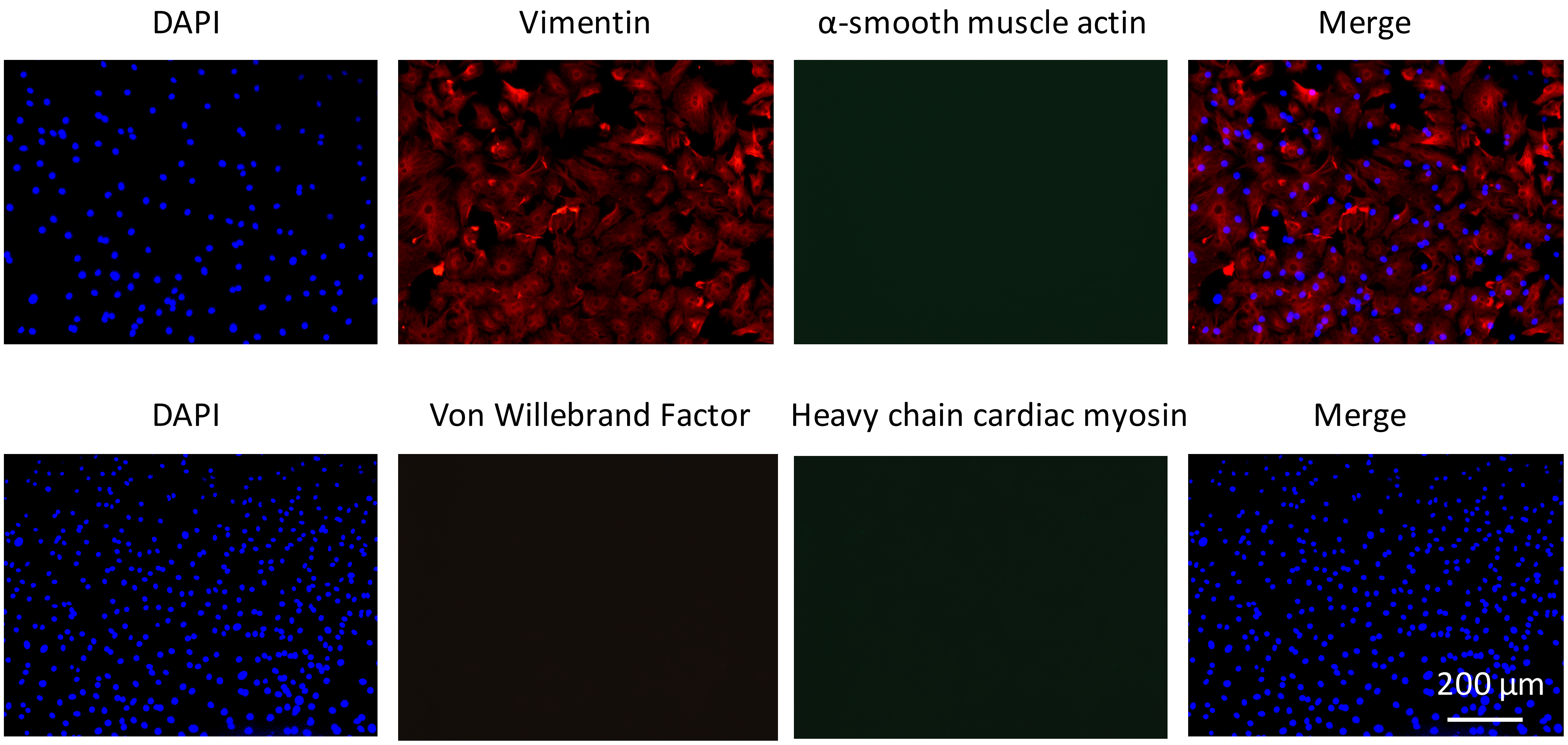

Supplemental Figure S2. (A) Representative of mitochondrial morphology in RV fibroblasts with or without P110 treatment for 24h.

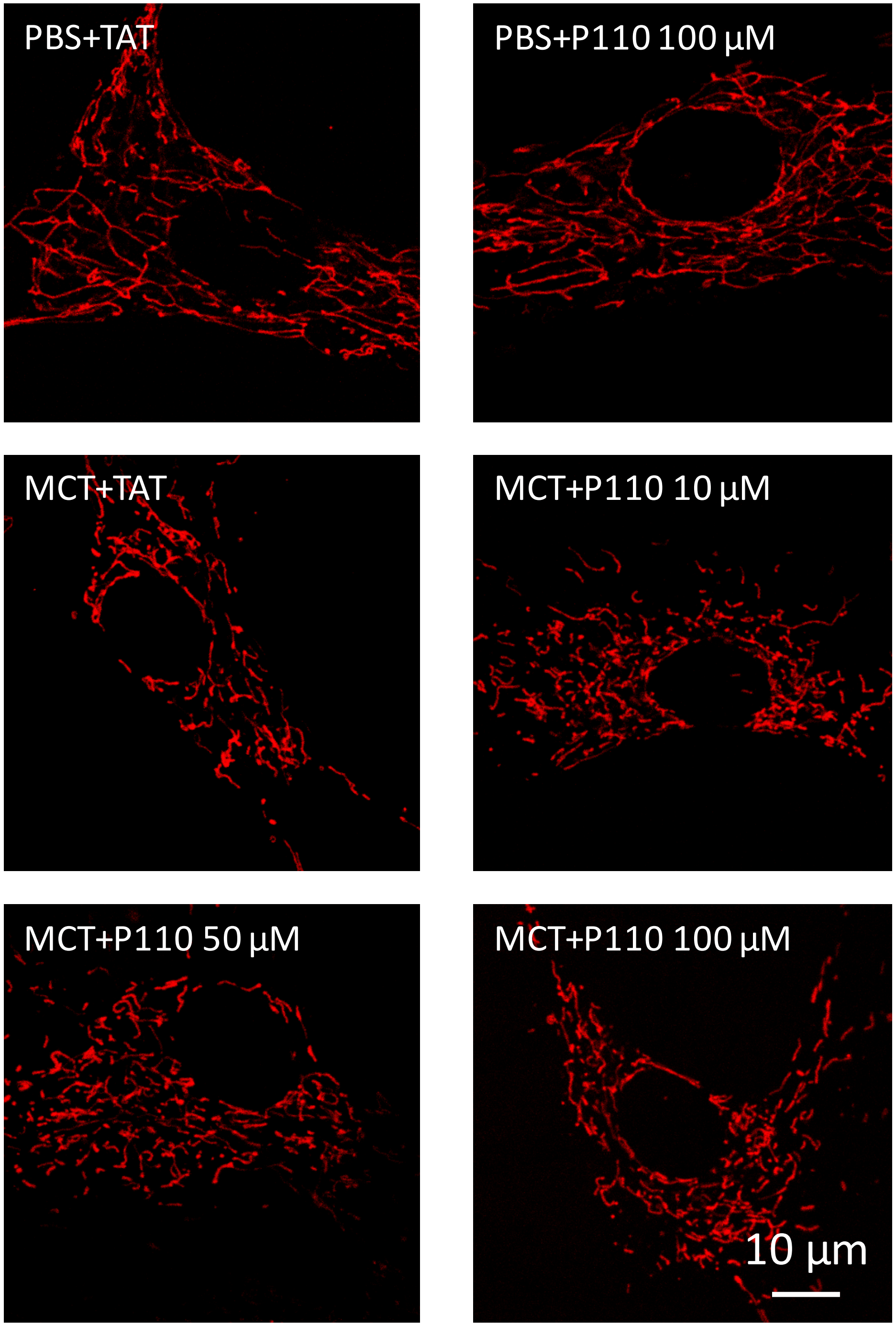

Supplemental Figure S2. (B) Representative of mitochondrial morphology in RV fibroblasts with or without P110 treatment for 48h.

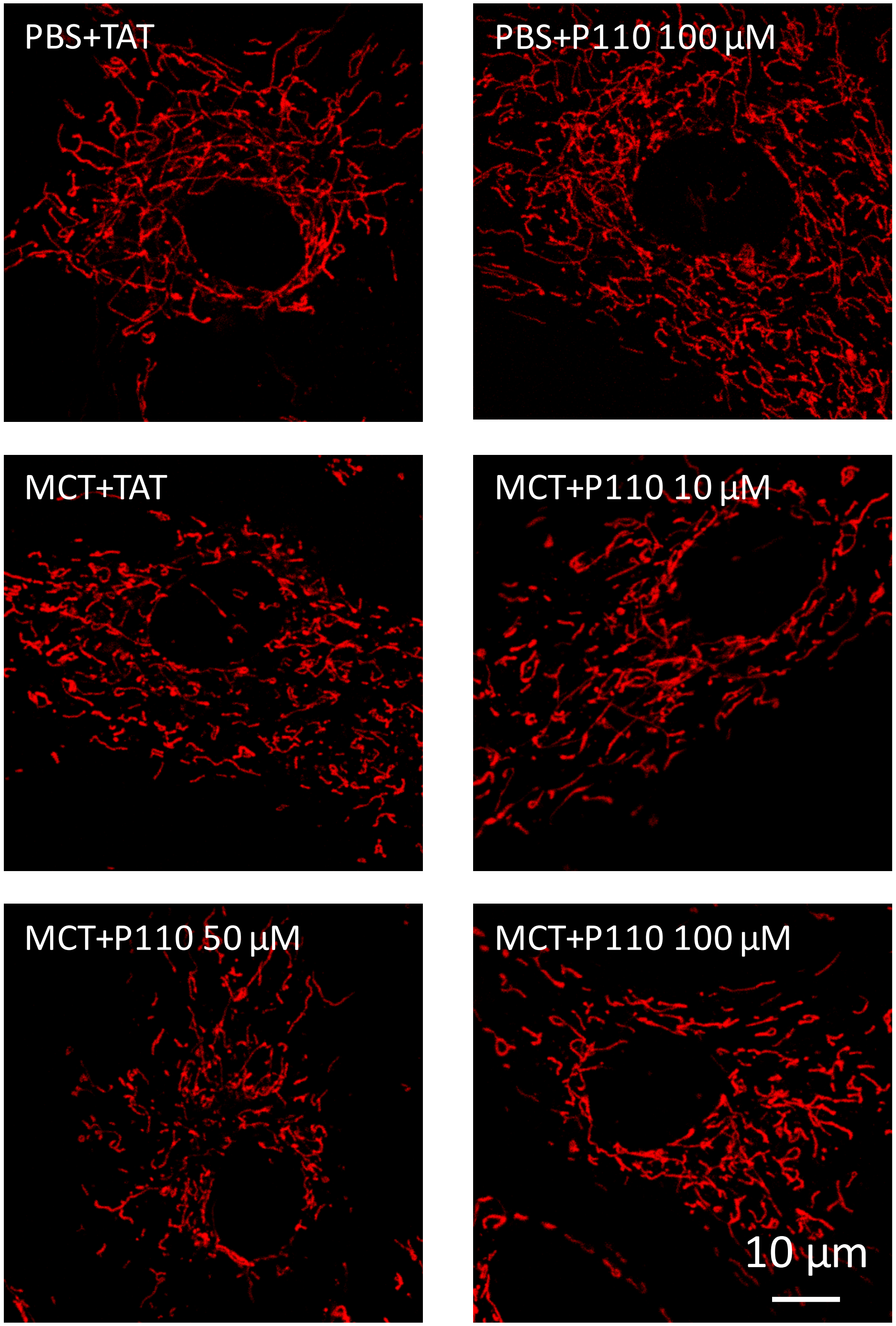

Supplemental Figure S3. mRNA expression of fusion mediators, mitofusin-1 (MFN1), mitofusion-2 (MFN2) and optic atrophy 1 (OPA1), are not changed significantly in monocrotaline (MCT)-induced RV fibroblasts versus the control (i.e., PBS group), and there is a trend toward reduction in MFN1 (P = 0.21) and MFN2 (P = 0.32). n=6~8 per group.

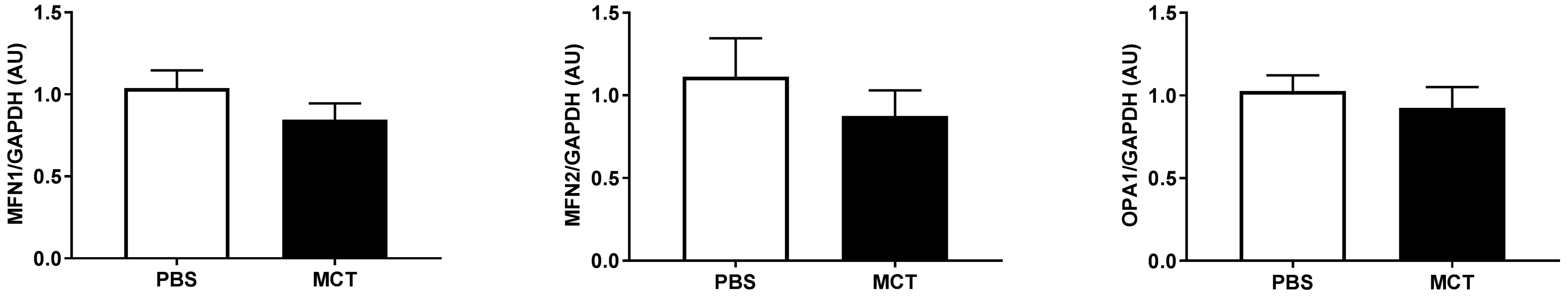

Supplemental Figure S4. Immunoblotting shows a trend toward reduction (P = 0.18) in mitofusion-2 (MFN2) in monocrotaline (MCT)-induced RV fibroblasts versus the control (i.e., PBS group). n=4~6 per group.

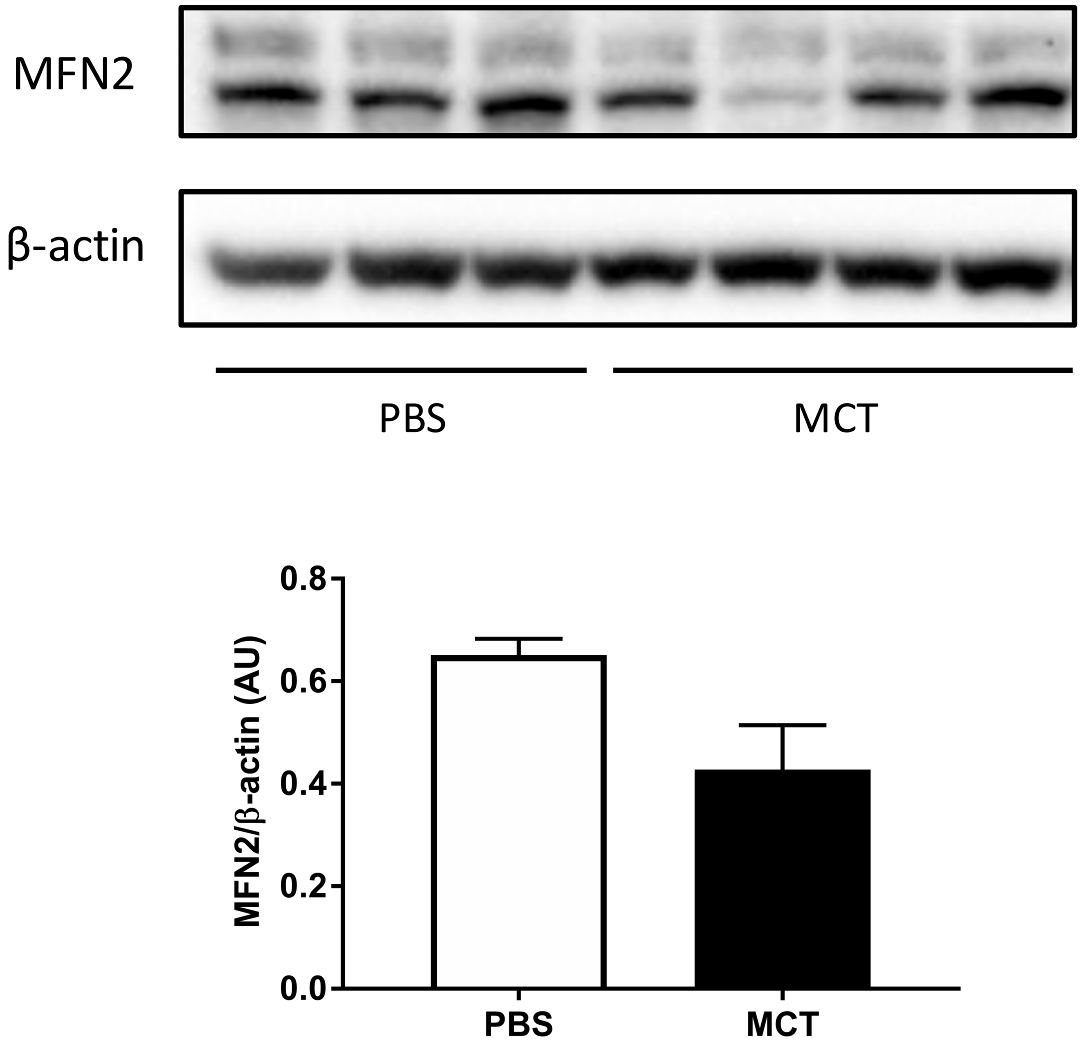

Supplemental Figure S5. Monocrotaline (MCT) reduced body weight of rats and P110 had no effect on body weight. MCT-T1 and MCT-P1, MCT rats treated with TAT and P110 respectively on day 14 and 19; MCT-T2 and MCT-P2, MCT rats treated with TAT and P110 respectively every other day starting from Day 10; MCT-P3, MCT rats treated with P110 only once immediately before the injection of MCT. ***, $P < 0.001$ versus PBS group. n=4~10 per group.

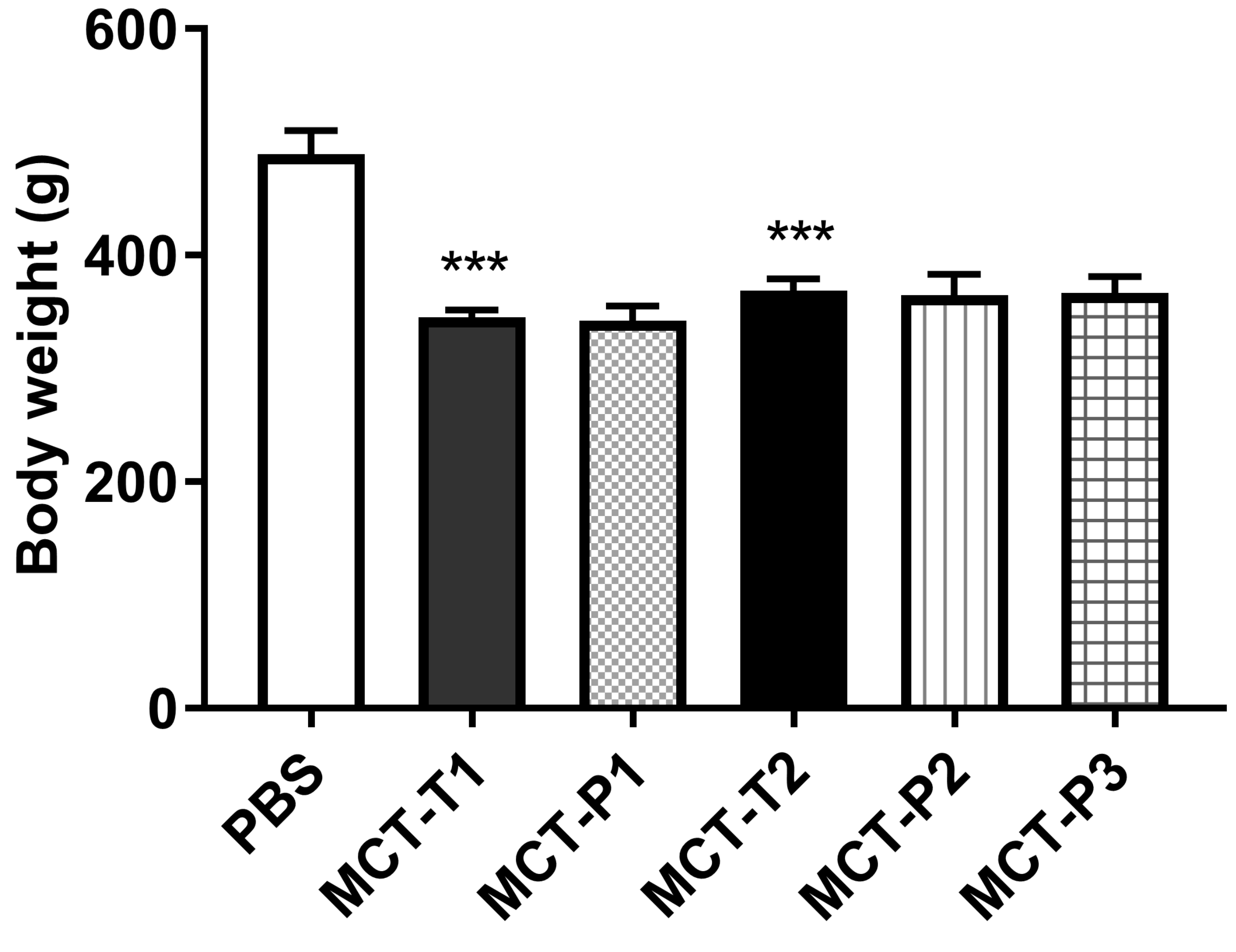

Supplement: Supplementary file 1 [file Presentation_1.pdf]
